# Comparing Australian orthopaedic surgeons’ reported use of thromboprophylaxis following arthroplasty in 2012 and 2017

**DOI:** 10.1186/s12891-019-2409-3

**Published:** 2019-02-08

**Authors:** Corinne Mirkazemi, Luke R. Bereznicki, Gregory M. Peterson

**Affiliations:** Division of Pharmacy, School of Medicine, University of Tasmania, Private Bag 26, Hobart, Tasmania 7001 Australia

**Keywords:** Arthroplasty, Thromboprophylaxis, Venous thromboembolism, Survey, Guideline, Aspirin

## Abstract

**Background:**

It is generally accepted that all arthroplasty patients should receive venous thromboembolism (VTE) and bleeding risk assessments, and that postoperative thromboprophylaxis be routinely prescribed where appropriate. Guideline recommendations regarding what to prescribe, however, have been inconsistent over the years, particularly regarding the appropriateness of aspirin. Our aim was to explore thromboprophylaxis patterns in use following hip and knee arthroplasty in Australia, and to examine associated variables.

**Methods:**

Orthopaedic surgeons were invited via mail to participate in two national surveys, conducted in 2012 (*N* = 478) and 2017 (*N* = 820), respectively.

**Results:**

The final response rates were 50.0 and 65.8% for 2012 and 2017, respectively. The thromboprophylaxis prescribing routines reported by respondents were divided into four categories: *anticoagulant-only* (the same anticoagulant-only routine for everyone), *aspirin-only* (aspirin for everyone), *staged-supply* (an anticoagulant during the initial postoperative period, followed by aspirin, for everyone) and *risk-stratification* routines (differing regimens depending on patients’ perceived risk of VTE). The most common approaches reported were anticoagulant-only routines; however, their popularity almost halved within the five-year period (from ~ 74% to ~ 41%). Conversely, staged-supply and risk-stratification protocol usage increased by more than two and nine times, respectively. In 2017, over one-half of surgeons reported prescribing aspirin in their practice. Reported concern for postoperative VTE and infections (OR 0.555 95% CI 0.396–0.779, *p* = 0.001 and OR 1.455 95% CI 1.010–2.097, *p* = 0.044 respectively), as well as Arthroplasty Society membership (OR 2.814 95% CI 1.367–5.790, *p* = 0.005) were predictors for use of aspirin (Cox and Snell R square = 0.072). The factor most commonly reported to shape surgeons’ protocols was research literature. Factors limiting prescribing of pharmacological prophylaxis included a perception that it increases bleeding and wound infection risk, is inconvenient, and lacks evidence applicable to real-world practice.

**Conclusions:**

VTE prevention post-arthroplasty is an evolving and multi-faceted entity, influenced by a range of factors and seemingly in need of robust evidence from large clinical trials to guide practice. The data highlighted potential short-falls in practice related to aspirin over-use, which could be further explored and addressed in future studies in order to optimise patient outcomes and reduce the significant morbidity and healthcare costs associated with VTE following these increasingly common surgical procedures.

**Electronic supplementary material:**

The online version of this article (10.1186/s12891-019-2409-3) contains supplementary material, which is available to authorized users.

## Background

Hip and knee arthroplasties are increasing worldwide. In Australia [1, 2] and the United Kingdom (UK) [3], annual procedure numbers almost doubled and more than tripled between 2003 and 2016, respectively. Although comparable data is limited, similar trends have also been described in the United States of America (USA) [4, 5]. These trends have potentially significant repercussions given that both procedures are considered risk factors for a leading cause of death and disability worldwide, namely venous thromboembolism (VTE) [6].

To reduce the risk of VTE post-arthroplasty, it is generally agreed that following individual VTE and bleeding risk assessments, postoperative thromboprophylaxis should be routinely prescribed for all patients. Guideline recommendations regarding what to prescribe, however, have been at variance with one another over the years, particularly regarding the appropriateness of aspirin as a thromboprophylactic agent [7–15]. The American Association for Orthopaedic Surgeons (AAOS) chose not to provide any recommendations for or against specific prophylactics in its guideline, however its Australian counterpart, the Arthroplasty Society of Australia (working under the auspices of the Australian Orthopaedic Association) has listed aspirin as an appropriate agent in at least the last three editions of its guideline, for patients at ‘low’ or ‘routine’ risk of VTE following arthroplasty [7–9, 15]. In 2012 the American College of Chest Physicians (ACCP) recommended aspirin as an appropriate prophylactic agent post-arthroplasty (with preference given to low molecular weight heparin over aspirin); this was in stark contrast to their previous 2008 guideline which specifically recommended against using aspirin as a sole agent [10, 11]. Until recently, the UK’s National Institute for Health and Care Excellence (NICE), like the Australian National Health and Medical Research Council (NHMRC), recommended against the use of aspirin post-arthroplasty [12, 13]. In the recent NICE guideline, however, there are recommendations for its use both following initial anticoagulant use (for hip arthroplasty patients) and alone (for knee arthroplasty patients), with no specific advice regarding when to use an aspirin-inclusive regimen over an anticoagulant-only one or vice versa [14]. These variances in recommendations regarding aspirin may inadvertently lead to either its over or under utilisation, thereby needlessly exposing patients to VTE and/or the risks associated with prophylactic and therapeutic anticoagulation.

We previously reported on a survey of Australian hip and knee surgeons examining thromboprophylaxis preferences, conducted in 2010 [16]. Given the varying and changing local and international recommendations regarding thromboprophylaxis post-arthroplasty, we have conducted two follow-up surveys, 5 years apart (in 2012 and 2017) to explore changes in the thromboprophylaxis patterns in use in Australia.

## Methods

The study was designed as a cross-sectional exploratory study, employing quantitative surveys conducted 5 years apart to meet its aims. The two surveys were conducted separately, each receiving ethics approval from the Tasmanian Social Science Human Research Ethics Committee.

### Study 1

An address list of Australian hip and knee surgeons was compiled by searching practice listings, hospital websites, and the internet. Invitations to participate were mailed to 478 surgeons in May 2012, with reminders sent two and 4 weeks later. Surgeons could complete an enclosed hard-copy or access the survey online; most participants completed hard-copies (95.9%).

### Study 2

The address list was updated using a similar search strategy, but also included searches of the online Royal Australasian College of Surgeons (RACS) *Find a Surgeon* directory (a directory of surgeons who voluntarily opt to have their contact details listed on the site). Invitations and surveys were mailed to 820 surgeons in June 2017, with reminders sent 3 and 7 weeks later. Given the prior preference for completing hard-copies, an online option was not provided.

The surveys were modified versions of a previously conducted survey, with both collecting data on respondents’ demographics, familiarity with guidelines, and practices and opinions regarding thromboprophylaxis and its efficacy [16]. The survey tool used in 2017 collected additional data regarding factors that influence surgeons’ practice, and surgeons’ level of concern regarding post-arthroplasty complications. They are available for viewing on the BMC Musculoskeletal Disorders website (Additional file 1: Appendix A and Additional file 2: Appendix B).

#### Statistical analysis

Data was analysed using SPSS (Statistical Package for the Social Sciences) 25.0 (IBM® Armonk, New York, USA). Continuous variables were summarised as means with standard deviations (SD). The differences between groups were tested using the t-test for independence and one-way analysis of variance with Tukey’s honest significant difference for continuous data, and chi-square test for categorical variables. Pearson’s rank coefficients were calculated for measuring correlations. Logistic regression analyses were conducted to assess which variables (such as: years practising, annual surgery load, sector of practice, ASA membership and reported level of concern for various postoperative complications) were most closely associated with surgeons’ preference to use or not use aspirin and other measures to minimise VTE in their practice, over and above mechanical and pharmacological prophylaxis. Multiple regression analyses were conducted to explore relationships between the level of concern reported by surgeons for different postoperative complications and other continuous variables (such as: years practising, annual surgery load and reported level of concern for other postoperative complications). Only *p* values ≤0.05 were considered statistically significant.

## Results

The final response rates were 50.0 and 65.8%, respectively (Table 1). Compared to Study 1, respondents in Study 2 conducted less arthroplasties, were less likely to be members of the Arthroplasty Society of Australia (ASA), and were less likely to conduct their clinical practice in the private sector.

**Table 1:** Surgeon responses and respondent demographics for Study 1 and Study 2

	Study 1 *2012*	Study 2 *2017*
Surgeons invited	478	820
Returned responses	257	596
Responses/Surgeons excluded due to …	36	165
...surgeon death / retirement / moving overseas	25	13
...invite being returned unopened	–	83
...surgeon not being a hip or knee surgeon	11	69
Surveys included (%) *N*	221 (50.0) *442*	431 (65.8) *655*
Male gender (%)	217 (98.6) *220*	416 (97.4) *427*
Years practising [mean (SD)] *n*	17.8 (9.0) *213*	17.6 (9.7) *424*
Sector of practice (%) **		
Private practice predominantly	155 (70.5)	247 (59.5)
Public practice predominantly	10 (4.5)	50 (12.0)
Both sectors equally	55 (25.0)	118 (28.4)
Relevant scope of practice (%)		
Hip only	3 (1.4)	3 (0.7)
Knee only	22 (10.0)	46 (10.7)
Both	194 (87.8)	381 (88.4)
Not specified	2 (0.9)	1 (0.2)
Annual arthroplasty load [mean (SD)] *n* *	175.2 (102.9) *215*	157.5 (108.6) *423*
Hip arthroplasties per year	76.3 (52.0) *193*	68.5 (57.8) *376*
Knee arthroplasties per year	108.3 (67.6) *212*	97.3 (71.2) *420*
ASA membership (%) *	48 / 209 (23.0)	62 / 425 (14.6)

* *p* < 0.05; ** *p* < 0.005; *ASA* = Arthroplasty Society of Australia; ASA members specialise in arthroplasty surgery and at least 80% of their surgeries must be joint replacements. *SD* = standard deviation

### Thromboprophylaxis routines and associated variables

#### Pharmacological preferences

The thromboprophylaxis prescribing routines reported by respondents were divided into four categories: *anticoagulant-only* (providing the same anticoagulant-only routine for everyone), *aspirin-only* (providing aspirin for everyone), *staged-supply* (providing an anticoagulant during the initial postoperative period, followed by aspirin, for everyone) and *risk-stratification* routines (providing differing regimens depending on patients’ categorisation as being either at ‘routine-risk’ or ‘high-risk’ of VTE). Most surgeons who performed both procedures preferred to use the same thromboprophylaxis prescribing routine type for both patient groups [191, 99.5%, *N* = 192 (Study 1); 364, 95.8%, *N* = 380 (Study 2)]. Many surgeons in both studies reported prescribing thromboprophylaxis beyond the initial hospital stay i.e. extended thromboprophylaxis therapy [168, 80.0%, *N* = 210 (Study 1); 384, 94.3%, *N* = 409 (Study 2)]. The mean reported duration of therapy was slightly higher for routine hip patients compared to knee patients [28.9, SD 14.1 days vs. 22.4, SD 15.1, *p* < 0.001 (Study 1); 36.6, SD 16.3 vs. 30.9, SD 16.9 days, *p* < 0.001 (Study 2)].

**Fig. 1:**
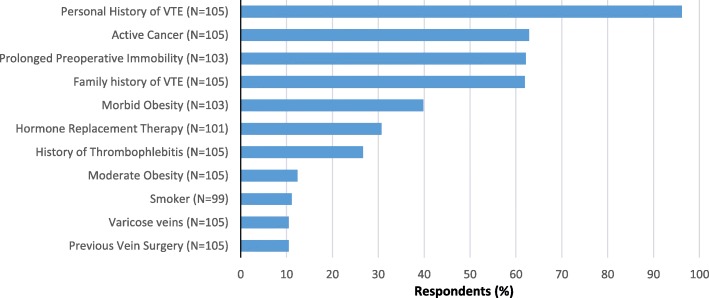
Factors classifying patients as ‘high-risk’ in risk-stratification protocols involving aspirin and anticoagulants (Study 2 only)

Excluding the Northern Territory, which only had two respondents, anticoagulant-only routines were the only protocol used throughout the country in 2012; in 2017, all except aspirin-only protocols were represented nationwide. While anticoagulant-only routines were the most popular approach in both studies (Table 2), their reported preference almost halved between surveys. Similarly, the number of surgeons who reportedly preferred to use aspirin-only routines in their practice, irrespective of their patients’ perceived VTE risk, also reduced between the two studies. However, the increase in the number of surgeons preferring risk-stratification protocols suggests there is an actual increase in the number of surgeons prescribing aspirin for patients. The most popular of the risk-stratification routines used aspirin for ‘routine-risk’ patients and an anticoagulant for ‘high-risk’ patients. The top three patient factors associated with being classified as ‘high-risk’ by these surgeons were a personal history of VTE, active cancer and prolonged preoperative immobility (Fig. 1). In contrast, most preferred aspirin in patients with preoperative infection (75, 72.8%, *N* = 103) or a high falls risk (90, 85.7%, *N* = 105).

**Table 2:** Pharmacological routine type preferences for hip and knee arthroplasty in Study 1 and Study 2

	Study 1 (*N* = 221)		Study 2 (*N* = 431)	
	Hip *n = 197 (%)*	Knee *n = 216 (%)*	Hip *n = 384 (%)*	Knees *n = 427 (%)*
Anticoagulant-only	144 (73.1)	161 (74.5)	155 (40.4)	176 (41.2)
Staged-supply	16 (8.1)	19 (8.8)	76 (19.8)	86 (20.1)
Risk-stratification	9 (4.6)	10 (4.6)	143 (37.2)	156 (36.5)
*Routine patients*: Aspirin only *High Risk patients*: Anticoagulant only	2 (1.0)	2 (0.9)	103 (26.8)	116 (27.2)
*Routine patients*: Anticoagulant, then aspirin *High Risk patients*: Extended anticoagulant therapy	1 (0.5)	1 (0.5)	24 (6.3)	28 (6.6)
*Routine patients*: Anticoagulant as inpatient only *High Risk patients*: Extended anticoagulant therapy	–	–	6 (1.6)	6 (1.4)
*Routine patients*: Aspirin only *High Risk patients*: Anticoagulant, then aspirin	–	–	1 (0.3)	1 (0.2)
Protocol unclear, however employs anticoagulants and aspirin in a risk-stratification protocol.	6 (3.0)	7 (3.2)	9 (2.3)	5 (1.2)
Aspirin-only	25 (12.7)	23 (10.6)	7 (1.8)	6 (1.4)
Miscellaneous^a^	–	–	2 (0.5)	2 (0.5)
Thromboprophylaxis protocol not reported	3 (1.3)	3 (1.4)	1 (0.3)	1 (0.2)

^a^NB: *Miscellaneous* incorporates surgeons whose reported practice did not fit into any of the other categories e.g. prescribing a non-steroidal anti-inflammatory agent other than aspirin

A quarter of respondents in Study 1 (54, 24.9%, *N* = 217) and over half in Study 2 (246, 57.1%, *N* = 431) reported using aspirin in their protocol, either in a staged-supply, risk-stratification or aspirin-only protocol; these surgeons are hereafter referred to as *aspirin-prescribers*. Reported level of concern for VTE and surgical site infections (SSI), as well as ASA membership were statistically significant predictors for use of aspirin (Cox and Snell R square = 0.072) in Study 2. Visual analogue scales (VAS) were used in the survey to explore respondents’ level of concern for postoperative complications, including VTE and SSI, with a score of 0 indicating they were *not concerned at all*, and a maximum score of 3 indicating they were *very concerned*. For every unit increase in the VAS score related to VTE concern (e.g. from 1 to 2), the odds of the surgeon being an aspirin-prescriber reduced by a factor of 0.555 (95% CI 0.396–0.779, *p* = 0.001). Conversely, for every unit increase in their score related to SSI concern, the odds of the surgeon being an aspirin-prescriber increased by a factor of 1.455 (95% CI 1.010–2.097, *p* = 0.044). Being an ASA member increased the odds of a surgeon being an aspirin-prescriber by a factor of 2.814 (95% CI 1.367–5.790, *p* = 0.005). There were no significant variables in Study 1. In 2012 only two surgeons specified a total daily aspirin dose (100 mg and 300 mg). In 2017 the most commonly specified total daily dose was 100 mg (120, 62.5%), followed by 150 mg (39, 20.3%), 300 mg (25, 13.0%), 200 mg (6, 3.1%), 75 mg (1, 0.5%) and 500 mg (1, 0.5%, *N* = 192).

**Fig. 2:**
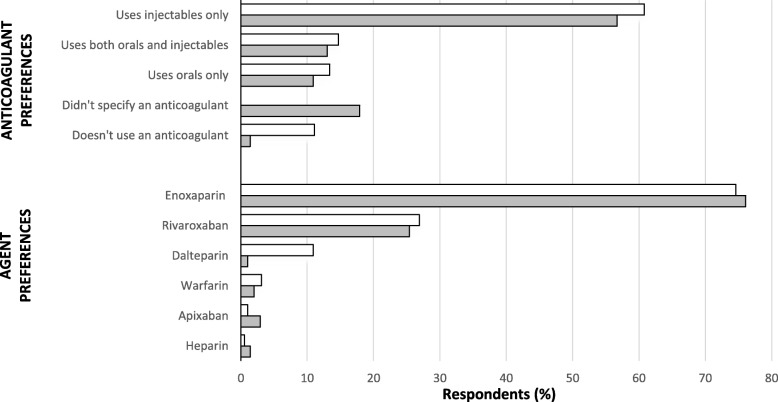
Anticoagulant and agent preferences (Study 1 (*N* = 217 and 193); Study 2 (*N* = 430, and 347). Legend:  Study 1;  Study 2

Most respondents reported using anticoagulants [193, 88.9%, *N* = 217 (Study 1); 424, 98.6%, *N* = 430 (Study 2)]; of these, most preferred injectable agents alone, and generally enoxaparin (Fig. 2). Regarding the dosing of anticoagulants, only 14 surgeons in Study 1 reported an actual dose, all of whom reported prescribing the recommended dose for thromboprophylaxis for each agent in Australia (i.e. apixaban 2.5 mg twice daily, dalteparin 2500 or 5000 units daily, enoxaparin 20 mg or 40 mg daily, rivaroxaban 10 mg daily etc.). In Study 2, most surgeons who disclosed their preferred anticoagulant agent(s) also reported a dose (336, 96.8%, *N* = 347), almost unanimously using either the recommended dose for thromboprophylaxis for the agent (315, 93.8%) or adjusting the dose to patients’ total body weight, lean body mass or body mass index (17, 5.1%, *N* = 336). Where reported, warfarin INR target ranges were either 1.5 to 2.0, 1.5 to 2.5, or 2.0 to 2.5 (4, 5.7%, *N* = 7).

#### Mechanical preferences (study 2 only)

Most surgeons reported using mechanical prophylaxis (403, 95.3%, *N* = 423). Of those with a stated preference (*N* = 376), compressive devices were most common (338, 89.9%), either alone (174, 51.5%) or with thromboembolic stockings (164, 48.5%, *N* = 338). Of these, calf compressive devices were the most popular (214, 63.3%, *N* = 338), used either alone (189, 88.3%) or in combination with venous foot pumps (25, 11.7%, *N* = 214). Aspirin-prescribers reported employing mechanical prophylaxis marginally more often than their counterparts (97.5% vs 92.2%, *p* = 0.018, *N* = 423), and tended to be more likely to prefer a compression device (92.5% vs. 85.9%, *p* = 0.053, *N* = 376).

### Other measures to minimise VTE risk (study 2)

Three hundred and forty surgeons reported taking measures over and above postoperative mechanical and pharmacological prophylaxis to minimise VTE risk post-arthroplasty (78.9%, *N* = 431), with early mobilisation being the most commonly reported measure (311, 91.5%, *N* = 340, Table 3). The logistic regression model explained only 5.7% of the variance (Cox and Snell R square). Aspirin-prescribers were more likely to report taking measures over and above mechanical and pharmacological prophylaxis (OR 1.834 95%CI 1.093–3.044, *p* = 0.006), as were surgeons who practiced predominantly in the private sector (compared to surgeons who practiced in the public sector either predominantly or equally, OR 2.558 95%CI 1.529–4.274, *p* < 0.001). The measures reported by respondents are outlined in Table 3 in three categories: those that occur pre-surgery, during surgery, and post-surgery. In addition to those listed, 10 surgeons (2.9%) reported avoiding operating on smokers (*n* = 9) and/or the morbidly obese (*n* = 5), three surgeons (0.9%) reported using an enhanced-recovery-after-surgery protocol, which typically involves components of care across all three care stages, [17] and five (1.5%) reported providing patients with VTE risk minimisation education, although it was unclear when this occurs (*N* = 340).

**Table 3:** Other measures reportedly used by surgeons to minimise VTE risk (*N* = 340)

Measure	Frequency (%)
Pre-surgery	
Prescribes exercise, weight loss, hydro and/or physiotherapy, and requires patients be smoke-free for 6 weeks prior to surgery	4 (1.2)
Avoids patients on HRT or ceases it pre-surgery	4 (1.2)
Admits patients on day of surgery	3 (0.9)
During surgery	
Regional anaesthesia	41 (12.1)
Avoids/minimises tourniquet use	24 (7.1)
Intra-articular anaesthesia	11 (3.2)
Intraoperative mechanical prophylaxis	9 (2.6)
Ensures minimal operation times	3 (0.9)
Intraoperative heparin	2 (0.6)
Avoids bilateral operations	1 (0.3)
Inferior vena cava filter (with warfarin) in high risk patients	1 (0.3)
Post-surgery	
Early mobilisation	311 (91.5)
Hydration	23 (6.8)
Ankle and bed exercises	20 (5.9)
Limb elevation	9 (2.6)
Early hospital discharge	3 (0.9)
Ensures ‘good’ postoperative analgesia	2 (0.6)

### Protocol-shaping factors (study 2 only)

Three hundred and nine surgeons (71.7%, *N* = 431) listed at least one factor that had shaped their thromboprophylaxis protocol, the most common of which was research literature (125, 40.5%, *N* = 309, Table 4). Two-fifths (28, 43.1%, *N* = 65) of those who cited bleeding, wound and/or infections, specifically referred to anticoagulants (e.g. ‘*excessive bleeding with enoxaparin and rivaroxaban’*).

**Table 4:** Factors reported to shape surgeons’ thromboprophylaxis protocols (*N* = 309)

Protocol-shaping factor	Frequency (%)
Research literature	125 (40.5)
Patient complications	78 (25.2)
*Bleeding, wound and/or infections*	*65 (21.0)*
*VTE*	*7 (2.3)*
*Fatal VTE*	*6 (1.9)*
Experience	84 (27.2)
Guidelines	48 (15.5)
Colleagues	39 (12.6)
Local Protocols	27 (8.7)
Meetings/Conferences/Lectures	26 (8.4)
Patient convenience and compliance concerns	18 (5.8)
Medico-legal concerns	16 (5.2)
Training	10 (3.2)
Increasing obesity	1 (0.3)

Although relatively few respondents listed guidelines as having influenced their protocol (*n* = 48), three times as many stated elsewhere that they used one (or more) in practice (*n* = 144, 55.4%, *N* = 260). The most commonly cited guideline was the ASA guideline (82, 56.9%), followed by the AAOS (34, 23.6%), NHMRC (17, 11.8%), ACCP (13, 9.0%), Australian and New Zealand Working Party (ANZWP, 5, 3.5%) and NICE (3, 2.1%) guidelines. A further 56 (21.5%) surgeons reported using an amalgamation of unspecified guidelines, 22 reported using a local hospital guideline (8.5%), and 38 specifically reported not using them (14.6%).

Surgeons who preferred risk-stratification protocols were more likely to report using the ASA, AAOS and/or ACCP guidelines in practice (43.6%) compared to surgeons who preferred staged-supply (31.3%), aspirin-only (16.7%) or anticoagulant-only (11.9%) protocols (*p* < 0.001). Surgeons who preferred using anticoagulant-only protocols were the most likely to report using the NHMRC, ANZWP and/or NICE guidelines in practice (9.7% vs. 0–2.4% for other protocols, *p* = 0.004). Surgeons who preferred using aspirin-only protocols were the most likely to categorically state that they did not use a guideline (33.3% vs. 3.4–11.4% for other protocols, *p* = 0.007).

### Guideline familiarity

**Fig. 3:**
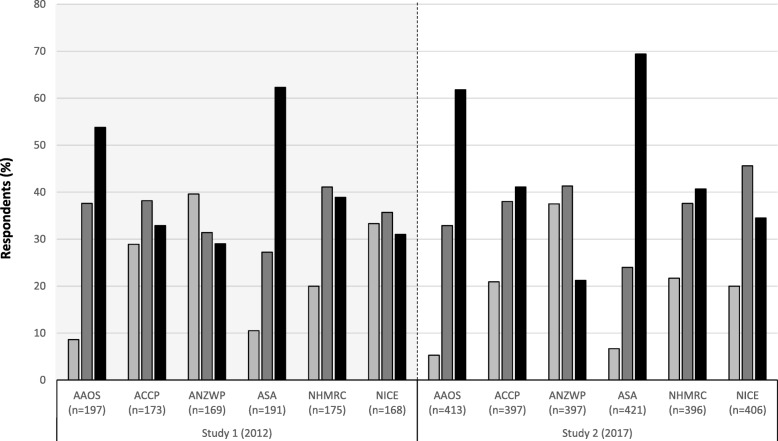
Guideline familiarity reported by respondents in Studies 1 and 2. Legend:  Not come across before;  Heard of in passing;  Very familiar with

Self-reported guideline familiarity was explored with respondents, irrespective of whether they reported using them or not. Over half reported being very familiar with orthopaedic-specific (AAOS and ASA) guidelines; similar high-level familiarity with multi-disciplinary guidelines was generally only reported by ≤40% of respondents (Fig. 3). Nevertheless, reported guideline familiarity broadly improved between studies. In Study 2, surgeons who reported being *very familiar* with the AAOS, ACCP, ANZWP, ASA or NHMRC guidelines were more likely to specifically report using that guideline compared with surgeons who marked that they had either *not come across* it or had only *heard of it in passing* (12.8% vs. 1.5% *p* < 0.001; 6.1% vs. 1.3% *p* = 0.009; 4.8% vs. 0.0% *p* = 0.001; 27.1% vs. 3.0% *p* < 0.001; 10.6% vs. 0.0% *p* < 0.001, respectively).

### Factors limiting pharmacological prophylaxis use

**Fig. 4:**
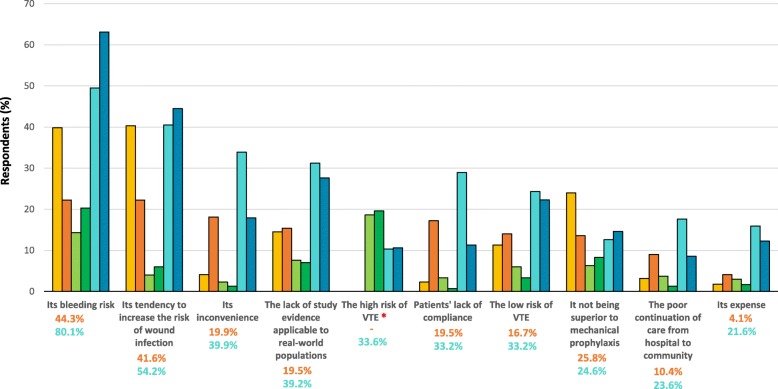
Factors limiting respondents’ pharmacological thromboprophylaxis use (Study 1 *N* = 221, Study 2 *N* = 301); *added in 2017. Legend:  Inpatient and  Discharge pharmacological prophylaxis use (Study 1);  Inpatient and  Discharge aspirin use (Study 2); Inpatient  and  Discharge anticoagulant use (Study 2). Total proportion of surgeons who answered the question in  and

In both studies, surgeons were asked to indicate if any factors limited their pharmacological prophylaxis use. In Study 2, this question addressed aspirin and anticoagulants separately. The top two factors were a perceived increased risk of bleeding and wound infections (Fig. 4). As rated on the VAS in Study 2 (0 = *not concerned at all* to 3 = *very concerned*), 89.8% of surgeons reported being concerned/very concerned about SSI, 70.5% about major bleeding, and 49.5% about minor bleeding; 80.2% said they were concerned/very concerned about patients developing postoperative VTE. These variables were statistically related in the multiple regression analysis. In particular, the biggest predictor for level of concern for VTE was reported level of concern for SSI (adj R square = 0.215, semipartial correlation coefficient = 0.216, *p* < 0.001), the biggest predictor for level of concern for SSI was reported level of concern for major postoperative bleeding (adj R square = 0.293, semipartial correlation coefficient = 0.322, *p* < 0.001) and the biggest predictor for level of concern for major postoperative bleeding was reported concern for minor bleeding (adj R square = 0.409, semipartial correlation coefficient = 0.317, *p* < 0.001), followed by reported level of concern for SSI (semipartial correlation coefficient = 0.295, *p* < 0.001). The number of years a surgeon had been practising was also a statistically significant predictor of level of concern for SSI (semipartial correlation coefficient = − 0.118, *p* = 0.004) and reported level of concern for major postoperative bleeding (semipartial correlation coefficient = 0.170, *p* < 0.001). There were no correlations with arthroplasty load.

**Fig. 5:**
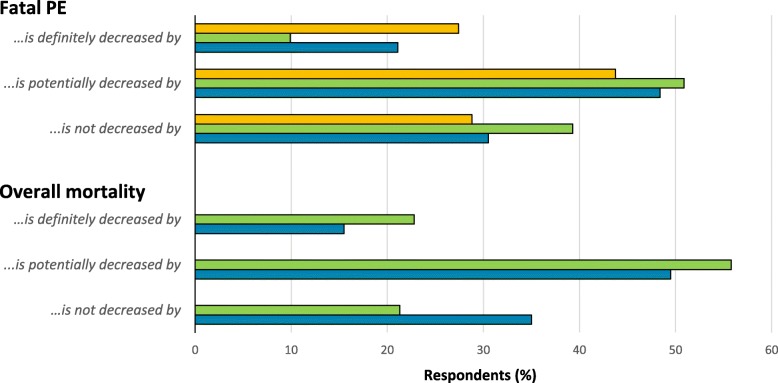
Surgeons’ opinions regarding pharmacological prophylaxis efficacy (Study 1, *N* = 208; Study 2, *N* = 403 to 413). Legend:  Pharmacological prophylaxis (Study 1);  Aspirin and  Anticoagulant prophylaxis (Study 2)

The third factor most commonly identified as limiting respondents’ pharmacological use post-arthroplasty was a perception that it is inconvenient, especially regarding anticoagulants in Study 2 (Fig. 4). Following closely behind was a perception that study evidence applicable to real-world practice is lacking. Correspondingly, less than half of respondents were confident that pharmacological prophylaxis was effective at reducing the incidence of fatal PE or overall mortality (Fig. 5). Aspirin-prescribers were more likely than their counterparts to believe aspirin was effective in preventing fatal PE (13.5% vs. 4.8%, *p* = 0.004) and overall mortality (30.7% vs 11.5%, *p* < 0.001), and were less likely to believe anticoagulants were effective [(16.9% vs. 26.6%, *p* = 0.021) and (12.1% vs 20.1%, *p* = 0.037)] (Study 2 data only). They were also more likely to report lacking study evidence as being a limiting factor to anticoagulant prescribing (43.7% vs. 23.1%, *p* < 0.001) compared to their counterparts, who were more likely to report it as being a limiting factor to aspirin use (6.1% vs 13.5%, *p* = 0.050).

## Discussion

Our surveys indicate that thromboprophylaxis practices following hip and knee arthroplasty are highly variable, and increasingly so, in Australia. Although the protocol type most commonly reported to be used in practice (anticoagulant-only) aligned with national recommendations at the time, our findings suggest that its popularity is declining. In its place, risk-stratification and staged-supply protocols are gaining momentum, neither of which were officially recommended at the time. Their rise in popularity and spread may be directly reflective of their increasing literature representation over the 5 years [18]. Interestingly, both protocols are recommended in some measure in the recent NICE guideline [14]. We anticipate that this – combined with the recent release of the EPCAT II study [19] results – will encourage their further widespread use.

Of note, at the time of writing, the evidence for aspirin-only protocols in hip arthroplasty patients specifically was limited, and they are not recommended in the recent NICE guideline [14, 18]. Nevertheless, almost one in three hip surgeons in our Study 2 cohort reported using aspirin-only routines in hip arthroplasty patients, either by employing a blanket approach or as part of a risk-stratification protocol. Furthermore, approximately one third of both hip and knee surgeons in Study 2 who said they preferred risk-stratification protocols did not consider active cancer or a personal history of VTE as factors that would classify their patient as being at high risk of VTE, and thus unsuitable for aspirin-only treatment. Together, these practices may be unnecessarily exposing patients to the risk of VTE and its associated treatment with therapeutic anticoagulation. Further, well-designed research investigating the efficacy and safety of aspirin-only routines in hip arthroplasty patients is needed to explore this more thoroughly.

That approximately 50% of surgeons in 2017 were using protocols which were not recommended at the time, but now are in some measure (i.e. risk-stratification and staged-supply protocols) [14], illustrates practice preceding guidelines. This is likely due to the laborious process of guideline production and its associated negative impact on the ability of professional bodies (esp. multi-disciplinary ones) to make rapid recommendations based on new data. In contrast, surgeons review and discuss research and conference papers as they are released, allowing greater speed and agility in implementing their findings. Correspondingly, the factor most commonly cited as having shaped respondents’ thromboprophylaxis practice was research literature. Although compared to Study 1, many more surgeons in Study 2 marked *a lack of study evidence applicable to real-world practice* as limiting their thromboprophylaxis prescribing, most of these responses related to anticoagulant use, with relatively few surgeons indicating that a lack of study evidence limited their aspirin use.

The second most commonly reported protocol-shaping factor was surgeons’ experience, particularly regarding postoperative complications. That so many more cited bleeding and wound issues, compared to VTE outcomes, is likely due to *‘bleeding being a much more readily apparent complication for the surgeon whose experience of VTE will be limited due to the overall low incidence of clinical VTE, and the majority of VTE events occurring after hospital discharge’* [20]. This limited exposure, particularly to fatal VTE, may explain why an increasing majority of surgeons in our study did not confidently believe that pharmacological prophylaxis prevents fatal PE. Furthermore, given its very low incidence, there is a shortage of clinical trials that are sufficiently powered to show statistically significant reductions in fatal PE. It is not surprising that surgeons are far from overwhelmingly convinced on the matter. Fatal PE is, however, only one cause of mortality following arthroplasty, and research suggests that it is not the leading cause; myocardial infarction is [21]. Given aspirin’s well-established role in the secondary prevention of cardiovascular disease, it is perhaps understandable why surgeons were more confident in the ability of aspirin to reduce overall mortality (compared to anticoagulants).

The third most commonly listed protocol-shaping factor was guidelines, and there were trends between surgeons’ preferred protocol and the guideline(s) they reported using in practice i.e. aspirin-prescribers were more likely to report using guidelines that accepted aspirin as an appropriate thromboprophylactic measure (38.6% vs. 12.4%, *p* < 0.001), and surgeons who preferred only using anticoagulants were more likely to report using a guideline that recommended *against* aspirin-use post-arthroplasty (9.7% vs. 1.6%, *p* < 0.001]). It is unclear, however, which factor influences the other i.e. are surgeons more likely to use a guideline that aligns with their prescribing preference, or are they more likely to prescribe a certain way based on the guideline they decide or are required to use? Further research is required to explore this.

A factor related to guideline use in Study 2 was guideline familiarity; surgeons were more likely to report using a guideline if they reported being familiar with it, suggesting that familiarity positively impacts uptake (although the reverse may also be true). It should be noted, however, that many surgeons who reported being *very familiar* with a guideline still did not report using it (72.9 to 93.9%, varying by guideline). Furthermore, our findings suggest that in comparison to the combined effects of research literature, experience and patient complications (listed by 52.9% of surgeons as having influenced their protocol), guidelines and local protocols appear to exert a quantitatively smaller influence (only listed by 16.2% of surgeons). Of all the guidelines reportedly used by surgeons, the orthopaedic-specific ASA and AAOS guidelines were the most popular. Compared to other guidelines, these both include pharmacological *and* non-pharmacological aspects of VTE prevention (over and above mechanical prophylaxis recommendations) in their main body of recommendations.

As with all research, the findings of these studies must be considered in light of their limitations. In particular, we only collected surgeons’ descriptions of their thromboprophylaxis practices, which may not accurately represent their actual real-world practices. Secondly, while our response rates were relatively high for this form of research, we cannot be certain that our invitation pools included all eligible surgeons in Australia. The combined annual arthroplasty load reported in our studies made up approximately 44.4 and 61.8% of surgeries recorded in the National Joint Replacement Registry in 2011 and 2016, respectively, and these proportions are similar to the respective response rates (50.0 and 65.8%, respectively) suggesting that the invitation pools were reasonably close to the actual eligible pool of hip and knee surgeons in Australia at the time [1, 2]. In the absence of being able to conduct a follow-up survey with non-responders to compare their views and practices with responders, we compared the responses from surgeons who responded to the first and third mail-outs in Study 2. There was no meaningful difference in their demographics, practices, or beliefs, suggesting that there is a good chance that the views and practices of our responders accurately reflect all surgeons conducting hip and knee arthroplasty in Australia.

## Conclusions

Our findings suggest that VTE prevention following hip and knee arthroplasty is an evolving and multi-faceted entity in Australia, with many different influencing factors and potentially some evidence-practice gaps that need further exploring. It will be interesting to observe whether the findings of the recently published EPACT II [19] study have an impact on practice and lead to less variation.

## Additional files

Additional file 1: Appendix A: Survey Tool used in 2012. (DOCX 38 kb)

Additional file 2: Appendix B: Survey Tool used in 2017. (DOCX 73 kb)

## Abbreviations

AAOS: American Association of Orthopaedic Surgeons
ACCP: American College of Chest Physicians
ANZWP: Australia and New Zealand Working Party
ASA: Arthroplasty Society of Australia
EPCAT II: The Extended Venous Thromboembolism Prophylaxis Comparing Rivaroxaban to Aspirin following Total Hip and Knee Arthroplasty II
NHMRC: National Health and Medical Research Council (of Australia)
NICE: National Institute for Health and Care Excellence
RACS: Royal Australasian College of Surgeons
SPSS: Statistical Package for the Social Sciences
SSI: Surgical Site Infections
VTE: venous thromboembolism
