# Supplementary material for: Comparing Australian orthopaedic surgeons’ reported use of thromboprophylaxis following arthroplasty in 2012 and 2017
Source: BMC Musculoskelet Disord. 2019 Feb 8;20:57. doi: 10.1186/s12891-019-2409-3 (PMC6368726; doi:10.1186/s12891-019-2409-3)
Supplement: Supplementary file 1 — Appendix A: Survey Tool used in 2012. (DOCX 38 kb) [file 12891_2019_2409_MOESM1_ESM.docx]

**Demographics**

**Are you a male or female?**

| - Male | - Female |
| --- | --- |

**How many years have you been qualified as an orthopaedic surgeon?** _______ years

**Are you a member of the Arthroplasty Society of Australia?** □ Yes □ No

**What state or territory do you practise in?**

| - ACT - NSW - NT | - Queensland - South Australia - Victoria | - WA - Tasmania |
| --- | --- | --- |

**Do you conduct most of your arthroplasties in the private or public sector?**

| - Private sector predominantly | - Public sector predominantly | - Both sectors equally |
| --- | --- | --- |

**Approximately how many hip and knee arthroplasties do you conduct in an average month?**

Hip Arthroplasty: ___________ per month

Knee Arthroplasty: ____________ per month

**Chemoprophylaxis Practises and Opinions**

**Do you believe that chemoprophylaxis (pharmacological thromboprophylaxis) decreases the rate of fatal PE?**

| - Not at all | - Potentially Decreases | - Definitely Decreases |
| --- | --- | --- |

**Do you prescribe chemoprophylaxis for your patients?**

| - Yes | - No |
| --- | --- |

*If yes*, what is your agent of choice? _________________________________________

Additionally, when do you prefer to stop chemoprophylaxis following each procedure?

Hip Arthroplasty: _____________________________________________________

Knee Arthroplasty: ____________________________________________________

**Please indicate if any factors below would prompt you to provide chemoprophylaxis for arthroplasty patients:**

| - Age < 70 yrs - Age ≥ 70 yrs - Current smoker - General anaesthesia - Hormone Replacement Therapy | - Moderate obesity (BMI > 30) - Morbid obesity (BMI > 40) - Preoperative infection - Previous VTE - Previous thrombophlebitis | - Previous vein surgery - Prolonged preoperative immobility - Surgery > 2 hrs duration - None of the above |
| --- | --- | --- |
| - Other ________________________________________________________ | | |

**Mark any of the following factors you feel limits chemoprophylaxis prescribing for arthroplasty patients:**

|  | **Inpatients** | **At discharge** |
| --- | --- | --- |
| It is not safe as it causes too much bleeding |  |  |
| It is not superior to mechanical methods |  |  |
| The risks of VTE are low |  |  |
| It is too expensive |  |  |
| It is too inconvenient |  |  |
| It increases the risk of wound infection |  |  |
| Study evidence is not applicable to real-world populations |  |  |
| Patients will not be compliant |  |  |
| There is poor continuation of care from hospital to community |  |  |
| None of the above |  |  |
| Other |  |  |

**Contemporary Guidelines**

**Mark which statement best fits the level of familiarity you have with each thromboprophylaxis guideline:**

|  | **very familiar** | **heard in passing** | **not come across** |
| --- | --- | --- | --- |
| ANZ Working Party |  |  |  |
| NHRMC |  |  |  |
| CHEST 9^th^ Ed |  |  |  |
| American Academy of Orthopaedic Surgeons |  |  |  |
| NICE Guideline (UK) |  |  |  |
| Arthroplasty Society Guidelines |  |  |  |

**In 2012 the CHEST physicians released their 9^th^ Ed. Guideline recommendations for VTE prevention:**

*“In patients undergoing major orthopaedic surgery, we recommend the use of one of the following rather than no antithrombotic prophylaxis: low-molecular-weight heparin; fondaparinux; dabigatran, apixaban, rivaroxaban; low-dose unfractionated heparin; adjusted-dose vitamin K antagonist; aspirin (all Grade 1B); or an intermittent pneumatic compression device (IPCD) (Grade 1C) for a minimum of 10 to 14 days.*

*We suggest the use of low-molecular-weight heparin in preference to the other agents we have recommended as alternatives (Grade 2C/2B), and in patients receiving pharmacologic prophylaxis, we suggest adding an IPCD during the hospital stay (Grade 2C). We suggest extending thromboprophylaxis for up to 35 days (Grade 2B). In patients at increased bleeding risk, we suggest an IPCD or no prophylaxis (Grade 2C). In patients who decline injections, we recommend using apixaban or dabigatran (all Grade 1B).”*

**Please outline any aspects of the CHEST 9^th^ Ed. recommendations that you feel are inappropriate.**

|  |
| --- |

**Please outline any aspects of the CHEST 9^th^ Ed. recommendations that you feel are appropriate.**

|  |
| --- |

**If you have any further comments on thromboprophylaxis prescribing following hip and knee arthroplasty,**

**or on the survey in general please feel free to leave them below or email** [ctaeed@utas.edu.au](mailto:ctaeed@utas.edu.au)**.**

|  |
| --- |

Enter your details below or email [ctaeed@utas.edu.au](mailto:ctaeed@utas.edu.au) if you would like to: □ receive study results □ enter iPad draw.

Name: _____________________________ Email: _____________________________
