# Supplementary material for: Comparing Australian orthopaedic surgeons’ reported use of thromboprophylaxis following arthroplasty in 2012 and 2017
Source: BMC Musculoskelet Disord. 2019 Feb 8;20:57. doi: 10.1186/s12891-019-2409-3 (PMC6368726; doi:10.1186/s12891-019-2409-3)
Supplement: Supplementary file 2 — Appendix B: Survey Tool used in 2017. (DOCX 73 kb) [file 12891_2019_2409_MOESM2_ESM.docx]

**Gender:** Male / Female  **Years practising as an orthopaedic surgeon:** ________ years

**Arthroplasty Society of Australia member?** *Yes / No* **State / Territory ______________**

**Do you conduct most of your arthroplasties in the private or public sector?**  Private / Public / Both equally

**Approximately how many hip and knee arthroplasties do you conduct in an average year?**

Hips: ___________ /year Knees: ____________ /year

**How concerned are you about your patients developing the following complications post-arthroplasty?**

|  | Not concerned at all | | Slightly concerned | | Concerned | | Very concerned | |
| --- | --- | --- | --- | --- | --- | --- | --- | --- |
| **VTE** |  | | | | | | | |
|  |  |  | |  | |  | |  |
|  |  |  | |  | |  | |  |
|  |  | | | | | | | |
| **Surgical Site Infection** |  | | | | | | | |
|  |  |  | |  | |  | |  |
|  |  |  | |  | |  | |  |
|  |  | | | | | | | |
| **Bleeding** |  | | | | | | | |
|  | **(minor)** |  | |  | |  | |  |
|  |  |  | |  | |  | |  |
|  |  | | | | | | | |
|  |  | | | | | | | |
|  | **(major)** |  | |  | |  | |  |
|  |  |  | |  | |  | |  |
|  |  | | | | | | | |

**Do you routinely prescribe thromboprophylaxis for your arthroplasty patients?** *Yes / No*

If yes, what is your routinely used pharmacological (including *agent(s), dose* and *duration*) and mechanical protocol?

***If the below table (and questions on the following page) do not adequately capture the subtleties of your protocol, please either ignore the lines in the table or attach your protocol as a separate page.***

|  | **Hip Arthroplasty** | **Knee Arthroplasty** |
| --- | --- | --- |
| ***Medicine(s):*** |  |  |
| **Agent** |  |  |
| **Dose** |  |  |
| **Duration** |  |  |
| ***Mechanical:*** |  |  |

**What other measures have you adopted to minimise VTE risk?**

______________________________________________________________________________________________

______________________________________________________________________________________________

**What factors have influenced your protocol?**

______________________________________________________________________________________________

______________________________________________________________________________________________

**Mark which description best fits the level of familiarity you have with each thromboprophylaxis guideline:**

|  | **very familiar** | **heard in passing** | **not come across** |
| --- | --- | --- | --- |
| ANZ Working Party 4^th^ Ed. |  |  |  |
| NHRMC (2009 – rescinded in 2016) |  |  |  |
| CHEST 9^th^ Ed (2012) |  |  |  |
| American Academy of Orthopaedic Surgeons (2011) |  |  |  |
| NICE Guideline (UK) (2015) |  |  |  |
| Arthroplasty Society Guidelines (2016) |  |  |  |

**Which guideline do you use in practice (if any)?_____________________________________________________**

**Please indicate if any factors below would prompt you to use aspirin or an anticoagulant post-arthroplasty:***

**Mark as few or as many as you deem applicable; if none, leave the row or table blank.*

|  | **Aspirin** | **Anticoagulant** |  |  | **Aspirin** | **Anticoagulant** |
| --- | --- | --- | --- | --- | --- | --- |
| Age < 70 years |  |  |  | Morbid obesity (BMI≥40) |  |  |
| Age ≥ 70 years |  |  |  | Preoperative infection |  |  |
| Cancer (active) |  |  |  | Previous vein surgery |  |  |
| Family history of VTE |  |  |  | Previous VTE |  |  |
| Current smoker |  |  |  | Previous thrombophlebitis |  |  |
| Creatinine clearance  ≤ 30mL/min |  |  |  | Previous surgical site infection |  |  |
| Hormone Replacement Therapy |  |  |  | Prolonged preoperative immobility |  |  |
| High falls risk |  |  |  | Regional anaesthesia |  |  |
| General anaesthesia |  |  |  | Surgery > 2 hrs |  |  |
| Moderate obesity (BMI≥30) |  |  |  | Varicose veins |  |  |
| Other(s) | | | | |  |  |

**Please indicate if any of the factors below limit your use of aspirin or an anticoagulant post-arthroplasty:***

**Mark as few or as many as you deem applicable; if none, leave the row or table blank.*

|  | **Aspirin** | | **Anticoagulant** | |
| --- | --- | --- | --- | --- |
|  | *Inpatient* | *Discharge* | *Inpatient* | *Discharge* |
| Its bleeding risk |  |  |  |  |
| It being not superior to mechanical methods |  |  |  |  |
| The *low* risk of VTE |  |  |  |  |
| The *high* risk of VTE |  |  |  |  |
| Its expense |  |  |  |  |
| Its inconvenience |  |  |  |  |
| Its tendency to increase the risk of wound infection |  |  |  |  |
| The lack of study evidence applicable to real-world populations |  |  |  |  |
| Patients’ lack of compliance |  |  |  |  |
| The poor continuation of care from hospital to community |  |  |  |  |
| Other(s) |  |  |  |  |

**In your opinion, how effective are aspirin and anticoagulants at decreasing fatal PE and overall mortality post hip and knee arthroplasty?**

|  |  | **Not effective at all** | **Potentially decreases** | **Definitely decreases** |
| --- | --- | --- | --- | --- |
| **Fatal PE** | *Aspirin* |  |  |  |
|  | *Anticoagulant* |  |  |  |
| **Overall mortality** | *Aspirin* |  |  |  |
|  | *Anticoagulant* |  |  |  |

*Thank you for taking your time to assist me with this survey.* If you have any further comments on VTE prevention following hip and knee arthroplasty, or on the survey in general please attach them as a separate page, or email [corinne.mirkazemi@utas.edu.au](mailto:corinne.mirkazemi@utas.edu.au) (if you would like the study results, please email me via this address too).
